# A call for an international network of genomic observatories (GOs)

**DOI:** 10.1186/2047-217X-1-5

**Published:** 2012-07-12

**Authors:** Neil Davies, Chris Meyer, Jack A Gilbert, Linda Amaral-Zettler, John Deck, Mesude Bicak, Philippe Rocca-Serra, Susanna Assunta-Sansone, Kathy Willis, Dawn Field

**Affiliations:** 1 Gump South Pacific Research Station, University of California Berkeley, BP 244 98728, Moorea, French Polynesia; 2 Biodiversity Institute, Department of Zoology, University of Oxford, The Tinbergen Building, South Parks Road, Oxford, OX1 3PS, UK; 3 Department of Invertebrate Zoology, National Museum of Natural History, Smithsonian Institution, PO Box 37012, MRC-163, Washington, DC, 20013, USA; 4 Institute for Genomic and Systems Biology, Argonne National Laboratory, 9700 South Cass Avenue, Argonne, IL, 60439, USA; 5 Department of Ecology and Evolution, University of Chicago, 5640 South Ellis Avenue, Chicago, IL, 60637, USA; 6 The Josephine Bay Paul Center for Comparative Molecular Biology and Evolution, Marine Biological Laboratory, Woods Hole, MA, 02543, USA; 7 Centre for Ecology & Hydrology, Maclean Building, Benson Lane, Crowmarsh Gifford, Wallingford, Oxfordshire, OX10 8BB, UK; 8 Oxford e-Research Centre, University of Oxford, 7 Keble Road, Oxford, OX1 3QG, UK

**Keywords:** Ecogenomics, Earth observation, Biodiversity, Ecosystems, Biocode, Genomic observatory, DNA

## Abstract

We are entering a new era in genomics–that of large-scale, place-based, highly contextualized genomic research. Here we review this emerging paradigm shift and suggest that sites of utmost scientific importance be expanded into ‘Genomic Observatories’ (GOs). Investment in GOs should focus on the digital characterization of whole ecosystems, from all-taxa biotic inventories to time-series ’omics studies. The foundational layer of biodiversity–genetic variation–would thus be mainstreamed into Earth Observation systems enabling predictive modelling of biodiversity dynamics and resultant impacts on ecosystem services.

## Review

Cosmologists have estimated that 95 % of the universe is dark matter or energy whose nature is still unknown. Here on Earth, there are so many species and genes about which we know little or nothing that estimates of total biodiversity could be off by orders of magnitude. Some 1.2 million species are scientifically catalogued but the vast majority of life on Earth still exists as “dark taxa”, with perhaps 86 % of extant eukaryotic species awaiting even the most basic description [1]. Yet it is now technically possible to sequence at least one gene from every macro (>1 mm) eukaryotic species in an ecosystem, as is already being done in the Moorea Biocode Project [2]. With sequencing costs falling at a rate outstripping even Moore’s Law [3,4], we can even begin to contemplate sequencing the whole genome of most macro eukaryotes in ‘model ecosystems’. For example, 10,000 genomes would cover all known (non-microbial) species on the island and coral reefs of Moorea–the number of genomes already called for by the Genomes 10 K Project for vertebrate species [5]. Similarly, we are now able to use new metagenomic technologies to elucidate long-term patterns of complex microbial communities, as has already been demonstrated by the Western Channel Observatory (L4) in the UK [6-11]. Indeed, as the ‘genomic revolution’ gains momentum we should start imagining a world where the biodiversity of key scientific sites is comprehensively documented at the genetic level. Such work would complement global taxonomic initiatives, such as the recent call to describe 10 million species in less than 50 years [12]. What we learn from pioneering place-based genomic research efforts will reduce global costs by developing best practice and testing new techno-logies. It will also help evaluate the benefits (for science and broader society) of describing the various dimensions of biodiversity, guiding approaches (e.g., identifying the best indicators), and helping prioritize outside the focal research sites. What will it take to achieve these goals and what will this vast new body of data tell us about our planetary life support systems? Here we address this question and argue for a coordinated effort to develop genomic observatories at intensively studied ‘research hotspots’ around the world.

### Next generation genomics

Biodiversity is generally defined as the variation found among genes, species, and ecosystems. The field of molecular biology has transformed our capacity to study living organisms at the genetic level, opening up whole new worlds for scientific exploration. Genetic material (DNA) is the common thread that unifies all life on Earth, with DNA forming an extraordinarily universal data type underlying all biodiversity. This remarkable consistency and ubiquity has facilitated innovation as engineers (and bioinformaticians) race to solve a clear challenge: building better and faster tools to ‘read’ DNA sequences.

Technological advances in DNA sequencing initially focused on the characterization of single genes, both from model organisms and environmental samples [13]. The genomic revolution started in 1995 with the first whole genome sequence of a bacterium [14,15]. We now have complete genomes for thousands of bacteria (including all major human pathogens) and hundreds of eukaryotes (including most model organisms) [16]. Interest in understanding the microbial make-up of diverse environments (e.g., ocean, soil, sediment, and a range of animal and plant ‘hosts’) combined with the development of ultra-high throughput sequence methodologies sparked a second revolution: the explosion of metagenomic studies sequencing the DNA of an entire community of organisms. Today, public DNA databases store more than 10^12^ bases of DNA from tens of thousands of studies (http://metagenomics.anl.gov). Megasequencing projects abound, characterizing hundreds if not many thousands of samples at a time. These include human populations [17], the Tree of Life [18], the International Census of Marine Microbes (ICoMM) and the world’s oceans [19,20], key sites of long-term study like the MIRADA-LTERS [21], and, most recently, from a myriad of global environments under the Earth Microbiome Project (EMP) [22]. Furthermore, as all organisms are evolutionarily related [23], access to their DNA and computational analyses of the differences among sequences has accelerated efforts to map the Tree of Life and stimulated initiatives like the International Barcode of Life project–a standardized DNA-based approach to species assignment [24].

Together, these advances in genomics have placed us on the cusp of the third major revolution: *exhaustive and sustained* sequencing of entire communities, and eventually whole ecosystems. Genomic data are relevant across multiple levels of biological organisation from Molecular Biology (genes, cells, and metabolic pathways), to Evolutionary and Developmental Biology (organisms and species), to Ecology and the Environment (populations, communities, and habitats). Rapid progress is now being made at all these levels permitting a *‘*new synthesis’ across the dimensions of biodiversity. Consequently, a unified systems approach to biocomplexity science (molecules-to-ecosystems) is within reach. To fully realize this potential, however, we argue that genomics needs to become more of a place-based science. Just as we have learned a great deal about general biological processes by applying genomics to a small number of model organisms in biomedical research, we can maximize advances in ecological understanding by focusing our sequencing efforts on already intensively studied model ecosystems.

### Imaging the biocode

The totality of genetic variation in a place at any given moment might be termed the “biocode” of an ecosystem, and we now have unprecedented tools for capturing these fundamental biodiversity data. Like taking a temperature, recording a genetic signature in space and time (measuring or ‘imaging’ the biocode) is fast becoming a relatively routine operation. DNA-level observations (sequences) are thus poised to become core components of future Earth Observing systems. For example, the Group on Earth Observations Biodiversity Observation Network (GEO BON) has already called for efforts to monitor and assess genetic diversity [25], while the Global Biodiversity Information Facility (GBIF) has predicted that the “currency of knowledge” pertaining to “the estimated 90 % of the planet’s biodiversity that is still to be discovered and shared… will not be phenotypic data, but primarily genomic biodiversity data, with identifiers linked to animals, plants, microbes, and ecosystems” [26].

If the opportunities for biodiversity genomics are clear and the swift pace of technological innovation impressive, at least three major challenges remain. First, despite the lower costs of sequencing, even small scale genomic studies are still expensive in terms of the time and resources required to annotate and interpret the vast quantities of resulting data. Second, although there are efforts to develop *in-situ* genomic sensors [27], field collection remains a relatively incompressible cost of ecogenomics (i.e., satellites cannot remotely sense DNA sequences, and physical collections require trained field scientists). Third, sequence data alone are of limited value without locating them in time and space and contextualizing them with other data (biological, ecological, environmental, and social). We believe that these constraints make a compelling case for consolidating ecogenomic efforts at inter-disciplinary research sites rich in contextual data produced by other long-term studies. In order to maximize scientific return on investment, therefore, we propose the establishment of Genomic Observatories (GOs) as a network of places (model ecosystems) that will serve as innovation incubators for ecogenomics. GOs will be sites (often supported by field stations and marine labs) that are equipped to pioneer the emerging science, engineering, informatics, and computing of ecogenomics; they will help evolve more mature solutions that are then capable of deployment in a much wider range of situations and places.

An observatory is a social construct–the “institutionalization of the act of observation”. Each genomic observatory will necessarily have its own characteristics, reflecting the diversity of the planet’s socio-ecosystems, the unlimited nature of scientific enquiry, and the plethora of emerging genomic technologies, informatics approaches, and analytical models. Nevertheless, all GOs will share two core goals: (i) to illuminate the dark taxa of biodiversity, and (ii) to sustain the sequencing of an entire ecosystem in the context of long-term biophysical and socioeconomic studies. The well-contextualized genetic sequences generated by GOs can be further ‘unpacked’, or re-annotated over time whenever new knowledge is acquired and as novel analytical tools are developed. Well-preserved and well-contextualized biomaterials (i.e., the physical samples), however, offer even more potential for future data driven discovery because additional sequences (and other types of molecular information) can be extracted as emerging technologies and lower costs permit.

We currently have but a fraction of the ’omic capability that will soon be broadly available. As prices fall and technologies advance, returning to historic biological samples will improve tomorrow’s models of Earth’s life support systems, enabling future generations to better manage the ecological consequences of rising greenhouse gas emissions and other drivers of change. GOs should also, therefore, consider how to archive and share biological samples in a way that maximizes their future utility for ’omic analyses. Crucially, any GOs bio-repository effort must take into account intellectual property concerns–notably the Access and Benefit Sharing (ABS) protocol [28] of the Convention on Biological Diversity (CBD)–particularly for samples that will be analysed using approaches not yet fully defined or even invented. We are launching a new service for GOs, entitled “International Ecostations”, which uses an e-journal infrastructure to help process applications and to publish ABS agreements. As publicly accessible and uniquely identified documents (e.g., using Digital Object Identifiers, DOIs), it will be easier to ensure that ABS agreements remain linked to a project’s downstream biomaterials and data products. Furthermore, leveraging citation services already developed by the scholarly publishing community (e.g., CrossRef.org) will enable upstream providers (including GOs) to better track the results of studies at their sites–a key demand from provider countries and a goal of the CBD. Other challenges for GOs include the sampling design (what to collect, where, and when) and practical issues of preservation techniques and economic sustainabi-lity. These issues must be confronted in collaboration with natural history museums, herbaria, and the biobank community who have experience providing stewardship for samples and vouchers. The effort will be worthwhile, as a time-series archive of biomaterials from GOs will be of great significance in helping to document changes through a potential “critical transition” [29] in Earth’s history–the geological epoch becoming known as the “Anthropocene” [30] where humans increasingly impact (even drive) Earth system processes.

### Towards a network of GOs

By focusing on places with rich histories of data collection and long-term commitments to future interdisciplinary studies, we will drive a scientific paradigm shift towards sustained sequencing of site-specific complex assemblages. Alongside their observatory functions, GOs are in prime positions to support numerous process-oriented studies and experiments, whether addressing local scale questions or as part of macro-system (continental/ocean scale) studies. At a technical level, the GOs network will serve as a test bed for a variety of innovative community-driven informatics solutions, as well as for trials of new generation sequencing technologies in a diverse array of settings and in pursuit of many different scientific questions. The commitment of scientific institutions to these sites offers added value through repeated sampling and contextualization of genomic time-series data. The concept of a living time-series (including extension into past and projection to the future) is particularly vital when we are considering the role of evolutionary as well as ecological forces in shaping past, present, and future socio-ecosystems. The inclusion of GOs at the best-characterized sites around the world, from the poles to the tropics, will allow an unprecedented view of life’s diversity and dynamics at its foundational layer, and it will support the assessment of differences along environmental gradients. Comprehensive sampling in a defined statistical framework will open up a world of comparative and computational studies within and between sites, enabling predictive modelling at the landscape scale [10,11]. While we are advocating greater coordination and a consolidation of effort at GOs, we do not mean to discourage genomic observations elsewhere. Indeed, the local models from GOs can be extended to make regional predictions, with data from less intensively studied areas providing crucial validation of the models. Similarly, high-resolution landscape scale data from GOs will help test and parameterize spatial biodiversity models developed from a range of different data types and at larger, regional scales [31,32]. We need a synergetic combination of data and models across multiple scales that serve to generate data (predictions) where no biodiversity observations have or can be made, ultimately providing complete images (continuous surfaces) of biodiversity for whole regions and eventually the planet [33].

Initial discussions with a number of sites and networks (see http://www.genomicobservatories.org) have demonstrated potential interest in expanding a GOs network beyond the sites with which we are associated: Moorea (ND, CM, LAZ) and L4 (JAG, DF) [34]. We aim for a series of international workshops to properly engage the wider community, catalyze and expand the global network of GOs, scope requirements for data integration, and build a shared website portal. In particular, efforts should be focused on the adoption of global data standards, common cyberinfrastructure, and shared informatics solutions that will ensure genetic data can be analyzed in context. Our approach places particular emphasis on surfacing genomic information using the recommended principles of shared standards [35] and “linked data”, such that they can be easily accessed, downloaded and integrated with other datasets (e.g., environmental and ecological) to create new analyses. In particular, the *Biodiversity Genomics Working Group*, a joint effort of the Genomic Standards Consortium (GSC) [36] and Biodiversity Information Standards (TDWG), will provide the GOs network with input from the major standards organizations in the genomics and taxonomic domains. The GOs should also work closely with the wider ISA Commons and BioSharing community [35,37]. In return, the GOs network represents a rich source of use cases (and users) for standards development. Finally, we envision the establishment of a new community, the *Biocode Commons*, bringing together developers, scientists, and standards organizations to provide the GOs Network with its primary forum for sharing resources, such as open source software tools that support genomic observations from collection through analysis and publication.

## Conclusions

In summary, the establishment of GOs at a subset of existing sites of major scientific importance will pave the way for taking the “biological pulse” of the planet. Thanks to DNA's remarkable uniformity and ubiquity, well-contextualized genetic data (like temperature and other meteorological measurements) are readily re-used across disciplines, institutions, and geographies. A network of GOs, equipped to generate and share DNA-level observations according to global data standards, would provide a powerful research infrastructure with which to address questions at the local level, cutting across habitat types and taxa, while also informing regional and global models. Long-term, DNA-centric, place-based work will go far beyond the sequencing of independent (unrelated, allopatric) genomes, to understanding the complete set of interactions of living organisms in a particular environment (ecosystem). Such an initiative must have a long-term (>30 year) vision. It should be built in collaboration with, and embedded within, existing networks like the International Long Term Ecological Research Network [38] and related initiatives (e.g., NEON [39], SI GEO [40], GEO BON [41], etc.). It will thus offer the promise of ‘accelerating returns’ by increasing our potential to characterize interaction networks and to address higher levels of organization. Such an approach would render tangible benefits to society through the enhanced ecosystem services expected from a better understanding of biodiversity dynamics. More information can be found at http://genomicobservatories.org.

## Abbreviations

EMP: Earth Microbiome Project; ILTER: International Long-Term Ecological Research Network; GBIF: Global Biodiversity Information Facility; GEOBON: Group on Earth Observations: Biodiversity Observation Network; GOs: Genomic Observatories; NEON: National Ecological Observatory Network; MIRADA-LTERS: Microbial Inventory Research Across Diverse Aquatic LTERS; SIGEO: Smithsonian Institution Global Earth Observatory.

## Competing interests

The authors declare that they have no competing interests.

## Author’ contributions

ND and DF wrote the first draft with subsequent input from CM, JAG, LAZ, JD, MB, PRS, SAS, and KW following discussions about the GOs Network concept. All authors read and approved the final manuscript.
